# Association of ABO Gene rs2073823 Polymorphism with Microvascular Complications, sP-Selectin Levels and Lipid Profile in Type 2 Diabetes

**DOI:** 10.2174/0115733998341750241208055924

**Published:** 2025-01-16

**Authors:** Haithem Rauf Mohammed, Rym Ben Othman, Zahraa Saad Hatef, Mohamed Kacem Ben Fradj, Haifa Abdesselem

**Affiliations:** 1 Faculty of Pharmacy, University of Monastir, Monastir, Tunisia;; 2 College of Medicine, University of Kerbala, Kerbala, Iraq;; 3 Faculty of Medicine of Tunis, University of Tunis El Manar, Tunis, Tunisia;; 4 Institut National de Nutrition et de Technologie Alimentaire de Tunis, service A, Tunis, Tunisia;; 5 Al Safwa University College, Karbala, Iraq;; 6 Laboratory of Biochemistry, LR99ES11, Rabta University Hospital, Tunis, Tunisia;; 7 Institut National de Nutrition et de Technologie Alimentaire de Tunis, service D, Tunisia

**Keywords:** Type 2 diabetes, ABO gene rs2073823 polymorphism, microvascular complications, diabetic retinopathy, sP-selectin, diabetic neuropathy

## Abstract

**Introduction:**

Type 2 diabetes (T2D) is a prevalent metabolic disorder linked to chronic inflammation and endothelial dysfunction, which contributes to the development of microvascular complications (MVCs) such as diabetic retinopathy (DR) and diabetic neuropathy (DN). Genetic factors, including variations in the ABO gene, may influence these complications. This study aimed to investigate the association between the ABO rs2073823 polymorphism and the risk of MVCs in patients with T2D, as well as its impact on inflammatory biomarkers, endothelial markers, and lipid profiles.

**Materials and Methods:**

We conducted an exploratory study involving 96 T2D Iraqi patients (Asian Arabic), examining the distribution of the ABO rs2073823 polymorphism and its correlation with MVCs. We assessed levels of inflammatory markers (TNF-α, IL-6, sE-selectin, sP-selectin), glycemic markers, renal function biomarkers, and lipid profiles. Adjustment was made for confounding factors including age, gender, body mass index, duration of diabetes, and hypertension.

**Results:**

Among the participants, 75% had MVCs, including DR (42%) and DN (65%). The ABO rs2073823 “A/A” genotype was associated with a reduced risk of MVCs under co-dominant (OR=0.16, *p*=0.045) and recessive models (OR=0.14, *p*=0.031). This protective effect remained significant after adjusting for confounding factors (OR=0.11, *p*=0.022). The “A/A” genotype was also linked to lower levels of total cholesterol, LDL-cholesterol, triglycerides, and sP-selectin. Patients with MVCs exhibited significantly higher levels of TNF-α, IL-6, and sP-selectin.

**Conclusion:**

The ABO rs2073823 polymorphism, particularly the “A/A” genotype, is associated with a decreased risk of MVCs in T2D patients and influences lipid metabolism and inflammatory markers. These findings suggest a genetic basis for the susceptibility to MVCs and highlight the role of the ABO gene in modulating inflammation and endothelial function in T2D. Further research is needed to validate these associations and explore potential therapeutic implications.

## 1 INTRODUCTION

Worldwide, just over half a billion people are living with diabetes [1]. Type 2 diabetes (T2D) is the most common type of diabetes, accounting for over 90% of all diabetes [1]. T2D is a non-communicable disease characterized by chronic metabolic derangements including hyperglycemia, defective insulin secretion, and insulin resistance [1, 2]. Moreover, T2D is associated with chronic inflammation (*e.g*, increased expression of proinflammatory cytokines such as TNF-α and interleukin IL-6) and endothelial dysfunction (*e.g*, increased expression of sE-selectin and sP-selectin) [3-7].

T2D patients are prone to develop diabetic-related microvascular complications (MVC) such as diabetic retinopathy (DR), diabetic neuropathy (DN), and diabetic nephropathy [8, 9]. MVC in T2D patients could lead to significant morbidity (*e.g*, vision impairment, blindness, end-stage renal, kidney, and cardiovascular diseases) that impair quality of life and increase the risk of mortality [8, 9].

Furthermore, chronic inflammation and endothelial dysfunction are implicated in the development of T2D and its MVC [2, 7, 10]. Chronic hyperglycemia, longer duration of T2D, hypertension, dysregulated blood lipid profile (high levels of triglycerides, total cholesterol, and low-density lipoprotein cholesterol), and insulin use are the most common risk factors associated with MVC [8, 9, 11]. In contrast, available evidence suggests that genetic susceptibility contributes to MVC [8, 12, 13]. Additionally, there is a polygenic architecture in determining MVC in T2D patients [12, 13]. In fact, multitudes of candidate genes associated with diverse MVC have been detected [12, 13].

The ABO gene is on the long arm of chromosome 9 (9q34.1-q34.2) and consists of 7 exons and 6 introns [14, 15]. Variation in the ABO gene is the basis of the ABO blood group, thus the presence of an allele determines the blood group in an individual [14, 15]. Furthermore, thousands of single-nucleotide polymorphisms (SNPs) in the ABO gene have been identified [16]. The ABO gene is a locus of considerable genetic diversity [17]. In fact, SNPs of the ABO gene play a pivotal role in the genetic etiology of many diseases such as pancreatic cancers [18], cardiocerebrovascular diseases [18], hypertension [19], COVID-19 [20] and type 1 diabetes [21]. Accumulated data suggested that ABO gene polymorphisms influence the individual susceptibility to the development of T2D [20, 21]. To our knowledge, data on the effect of genetic variation in the ABO gene and the risk of MVC are scarce.

In another part, the pleiotropic effects of the ABO gene have been demonstrated [22]. Pleiotropy is the phenomenon in genetics whereby a DNA variant influences multiple traits [23]. Several studies demonstrate that genetic variation in the ABO gene was responsible for the modulation of blood lipids profile and the variability of endothelial dysfunction and inflammatory biomarkers [22, 24-29]. The following factors (blood lipid profile, chronic inflammation, and endothelial dysfunction) were associated with MVC risk. By the way, these findings let us suppose that genetic variation in the ABO gene could modulate MVC risk.

In this context, the present study aimed to investigate the association between the ABO rs2073823 polymorphism and the risk of MVC in the Iraqi population. We also tested whether the ABO rs2073823 polymorphism could affect some traits like inflammatory biomarkers, endothelial markers, and blood lipids levels.

## 2 MATERIALS AND METHODS

### 2.1 Study Design and Participants

We enrolled in this study 96 unrelated T2D patients who underwent medical follow-up at Al-Hassan Center for Endocrinology and Diabetes (Karbala, Iraq) from April 2022 to March 2023. The T2D diagnosis was according to the American Diabetes Association (ADA) diagnosis criteria. MVC diagnosis was based on fundoscopic examination for DR, clinical assessments of symptoms and signs for DN, and estimated glomerular filtration rate (eGFR) in addition to serum creatinine levels for diabetic nephropathy. To be eligible to participate in the study, T2D patients must be aged between 30 and 70 years and be able to provide informed consent. Exclusion criteria were as follows: uncompleted medical files, presence of other chronic diseases than T2D such as cancer and renal or heart failure, and pregnancy. Data on demographic and clinical data, including age, gender, duration of diabetes, body mass index (BMI), and presence of hypertension, were collected through structured interviews and medical record reviews. The research ethics committee at the Faculty of Medicine of Karbala University approved the study. The study was conducted in accordance with the Declaration of Helsinki's statement of ethical principles for medical research involving human subjects. Lastly, written informed consent was obtained from each study participant after explaining the purpose and procedures of the study.

### 2.2 Biochemical Analyses

Blood samples were collected after eight hours of fasting for biochemical analyses in appropriate tubes for each biochemical test. Fasting blood glucose, HbA1c, total cholesterol, HDL-cholesterol, creatinin, and triglycerides were measured using standard enzymatic methods. eGFR was calculated using the modified MDRD formula. LDL-C estimation was assessed by the Friedewald equation. Inflammatory markers including TNF-α, IL-6, sE-selectin, and sP-selectin were quantified using enzyme-linked immunosorbent assay kits (Elabscience^®^, Elabscience Bionovation Inc., Houston, USA) according to the manufacturer's instructions.

### 2.3 Genotyping

DNA was extracted and purified using the gSYNC™ DNA extraction kit (Geneaid^®^, Geneaid Biotech Ltd., Taipei, Taiwan) following the manufacturer's instructions. DNA concentration and purity were assessed through absorbance measures of samples' microvolumes using a NanoDrop spectrophotometer. A tetra-primer amplification refractory mutation system PCR (ARMS-PCR) assay was used for the genotyping of the allele ABO rs2073823 (G/A). Four primers (2 allele-specific and 2 common primers) were used in this assay (Supplementary Table **1**). In order to maximize the specificity of allele-specific primers, a mismatch was introduced at the antepenultimate nucleotide of 3′ terminus. A 394 base pair (bp) fragment is an indicator of the “G“allele whereas a 262 bp fragment is an indicator of the “A” allele. To assess genotyping accuracy, a total of 25 DNA samples (25% of study participants) and 10 water blanks were genotyped by an independent clinician who was blinded to previously reported genotyping results. The assessed samples were all correctly genotyped.

### 2.4 Statistical Analysis

Statistical analysis was performed using SPSS statistical package version 27.0 (IBM SPSS Inc., New York, NY, USA) and GraphPad Prism version 9.0 (GraphPad Software, La Jolla, CA, USA). Comparisons between groups were assessed by independent-samples T test or one-way ANOVA test for continuous variables, as appropriate. A generalized linear model was used to test the association between biochemical parameters levels and ABO rs2073823 with adjustment for confounding factors including age, gender, body mass index, duration of diabetes, and hypertension. We noted that for continuous variables with non-normal distribution, a log-transformation was made before comparison. Normality of continuous variables was tested by the Shapiro-Wilk test. Only age and body mass index were normally distributed. The Hardy-Weinberg equilibrium was tested by a goodness-of-fit χ^2^ test. The chi-square test was used for categorical variables. We used binary logistic regression to test the association of the ABO rs2073823 polymorphism with MVC, DR, and DN risks, under co-dominant, dominant, recessive, and over-dominant genetic inheritance models. The Akaike information criterion (AIC) and the Bayesian information criterion. (BIC) was used to determine the best-fit model for ABO rs2073823. The intensity of the studied associations was estimated by crude and multi-adjusted odd-ratios with their 95% confidence intervals. The study is in conformity with the Guidelines of Strengthening the Reporting of Observational Studies in Epidemiology Statement for observational studies.

## 3 RESULTS

### 3.1 Demographic, Clinical, and ABO rs2073823 Polymorphism Data of Study Participants

Among the 96 patients with diabetes type 2 enrolled in this study, 40 patients (42%) had diabetic retinopathy (DR), 62 patients (65%) had diabetic neuropathy (DN), 4 patients had nephropathy (4.16%) and in total 72 patients (75%) had a microvascular complication (MVC). The genotype distribution of ABO rs2073823 polymorphism of study participants (“G/G” for 66 patients (68,7%), “G/A” for 24 patients (25%) and “A/A” for 6 patients (6.3%) was consistent with Hardy Weinberg equilibrium (*p*=0.09).

Table **1** summarizes the demographic and clinical characteristics of study participants in whole and stratified by MVC. Patients with MVC and patients without MVC differed only in term of age. In fact, patients with MVC were older than those without MVC. There were no significant differences between the two groups concerning other studied parameters including gender and hypertension distribution, body mass index, duration of disease, lipid profile as well as levels of biomarkers of renal function and glycemic markers.

Furthermore, demographic and clinical characteristics of study participants stratified by DR and DN were reported in supplementary Tables **2** and **3**, respectively. Patients with DR were older, had higher rate of hypertension, had lower levels of fasting blood glucose and HbA1c and had higher body mass index and levels of triglycerides compared to patients without DR. Patients with DN didn’t differ from those without DN by any studied demographic and clinical characteristics.

### 3.2 Association between Inflammatory Biomarkers and Microvascular Complications

Table **2** show levels of studied inflammatory marker (TNF-α, IL-6, sE-selectin and sP-selectin) of patients stratified by MVC, DR and DN. Generally, patients with microvascular complication (MVC, DR or NR) exhibit a significantly higher level of some studied inflammatory marker. Compared to patients without MVC, those with MVC had a significantly higher level of TNF-α, IL-6 and sP-selectin (Fig. **1**). Patients with DR had a significantly higher level of Cytokines (TNF-α, IL-6) (Fig. **2**) but not selectins (sE-selectin and sP-selectin) compared to patients without DR. Finally, patients with DN had a significantly higher level of cytokines IL-6 and sP-selectin in comparison to patients without DN (Fig. **3**).

### 3.3 Association between ABO rs2073823 Polymorphism and Risk of Microvascular Complications

Table **3** shows the genotypic distribution (under co-dominant, dominant, recessive, and over-dominant genetic inheritance models) and allelic frequency of the ABO rs2073823 polymorphism in patients with MVC and patients without MVC patients. The genotypic distribution of ABO rs2073823 polymorphism was significantly different between the two groups under the co-dominant model (*p*=0.039) and the recessive model (*p*=0.033). Un-adjusted and multi adjusted odds-ratios of MVC according to ABO rs2073823 genotypes and alleles were summarized in Table **3**. The ABO rs2073823 A/A genotype was associated with a decreased risk of MCV under the co-dominant model (OR (95%CI) A/A *vs.* G/G = 0.16 (0.27-0.96); *p*=0.045) and under the recessive model (OR (95%CI) A/A *vs.* (G/G+G/A) = 0.14 (0.02-0.84); *p*= 0.031). The recessive model had the lowest AIC and BIC values and thus it was considered as the best-fit model. The protective effect of ABO rs2073823 A/A genotype against MVC remains significant while adjusting on confounding variables including age, gender, body mass index, duration of diabetes, and hypertension (Multi-adjusted OR (95%CI) A/A *vs.* (G/G+G/A) = 0.11 (0.02-0.72); *p*= 0.022).

Supplementary Table **4** shows the genotypic distribution and allelic frequencies of ABO rs2073823 polymorphism in patients with DR and patients without DR patients. The allele frequencies were significantly different between the two groups. The “A” allele was associated with a reduced risk of DR. The genotypic distribution of ABO rs2073823 polymorphism was significantly different between the two groups under the co-dominant model (*p*=0.044), dominant model (*p*=0.044) and recessive model (*p*=0.039). The genotypes “G/A+A/A” under the dominant genetic model were associated with a reduced risk of DR but this association didn’t remain significant after adjustment for confounding factors. We noted that the “AA” genotype was detected in patients without DR but not in patients with DR and thus odds-ratio calculation was impossible. The over-expression of the AA genotype in patients without DR lets us assume that this genotype could be associated with a reduced risk of DR.

Supplementary Table **5** shows the genotypic distribution and allelic frequency of ABO rs2073823 polymorphism in patients with DN and patients without DN patients. There was no significant association between ABO rs2073823 polymorphism and risk of DN.

### 3.4 Association between ABO rs2073823 and Biochemical Markers

Variation in levels of biochemical markers (*i.e.* glycemic markers, biomarkers of renal function, inflammatory biomarkers and lipid profile) according to the ABO rs2073823 polymorphism under co-dominant and recessive genetic model were shown in supplementary Tables **4** and **6**, respectively. We found that the ABO rs2073823 polymorphism was significantly associated with the levels of total cholesterol, triglycerides and sP-selectin with a tendency of lowest value found in patients carrying the AA genotype.

Compared to patients carrying the GG+GA, patients carrying the AA genotype had a significantly lower levels of total cholesterol, LDL-cholesterol, triglycerides and sP-selectin but a significantly higher levels of HbA1c (Fig. **4**) even after adjustment for age, gender, body mass index, duration of diabetes and hypertension (Table **4**). Agarose gel electrophoresis of ARMS assay for ABO rs2073823 polymorphism revealed distinct bands: a 613 bp product for outer forward and outer reverse, with a 394 bp fragment indicative of the 'G' allele and a 262 bp fragment indicative of the 'A' allele (Fig. **5**).

## 4 DISCUSSION

In this study, we tested the association of the ABO rs2073823 polymorphism as well as selected inflammatory and endothelial dysfunction biomarkers, with the risk of MVC. We found an association between TNF-α, IL-6, sP-selectin and MVC. The ABO rs2073823 polymorphism “A” allele was associated with a protective effect against DR. Additionally, The ABO rs2073823 polymorphism “A/A” genotype was associated with a protective effect against MVC. It is noteworthy that the protective effect of the ABO rs2073823 polymorphism “A/A” genotype was independent of other MVC risk factors. Furthermore, we found that the ABO rs2073823 polymorphism modulates lipids profile and sP-selectin levels. We showed that T2D patients carrying the MVC-protective “AA” genotype had significantly lower levels of total cholesterol, LDL-cholesterol, triglycerides, and sP-selectin.

We found increased levels of studied cytokines (TNF-α and IL-6) and sP-selectin in MVC patients compared to patients without MVC. Our data were similar to previously reported results showing elevated levels of inflammatory and endothelial biomarkers in patients with MVC [30-33]. These findings confirm the recognized implication of chronic inflammation and endothelial dysfunction in these T2D complications [2, 7, 10].

Genetic variations in the ABO gene are associated with T2D risk and with other risk factors that modulate MVC (such as hypertension, chronic inflammation biomarkers, lipid profile, and markers of endothelial dysfunction) [19, 22, 24-29]. To our knowledge, data on the association of this ABO polymorphism and MVC risk were previously absent. In this study, we found for the first time an association between the ABO rs2073823 polymorphism and the risk of MVC. Previously, The ABO rs2073823 polymorphism was studied in a limited number of genetic association studies to test its correlation with hematologic traits [34, 35] and its association with glaucoma risk [36]. These studies revealed the valuable potential of this SNP. First, the ABO rs2073823 polymorphism, along with four other SNPs, was used to calculate a polygenic risk score that correlates with glaucoma risk. Second, the ABO rs2073823 polymorphism was associated with hematological traits [34, 35]. The ABO rs2073823 “A” allele was associated with decreased mean corpuscular volume (MCV) and elevated RBC counts in the Korean population [34] as well as with elevated hemoglobin levels and hematocrit indices in the Iranian population [35].

The ABO rs2073823 polymorphism is located in intron 6. It remains unexplored whether it could affect ABO gene functionality, such as the modulation of ABO m-RNA levels or splicing efficiency. Introns play an important role in gene regulation and expression, as well as in epigenetic regulations [37, 38]. Thus, SNPs located in introns could modulate disease risk by influencing RNA alternative splicing, genomic imprinting, gene expression through lncRNAs, transcription enhancers, chromatin looping, cell death programming, and premature stop codons [37, 38]. Unfortunately, there are currently no functional studies available that can definitively determine the plausible biological effect of this intronic polymorphism.

Alternatively, the ABO rs2073823 polymorphism could be non-functional but in linkage disequilibrium (LD) with another causal SNP. Therefore, the observed association between the ABO rs2073823 polymorphism and MVC risk could be attributable to LD with the causal SNP (an indirect effect) [39]. Data on SNPs in LD with the ABO rs2073823 polymorphism are still lacking. Currently, only the ABO rs8176746 has been found in strong LD with the ABO rs2073823 polymorphism [34].

Furthermore, associations between SNPs located in the intron areas of the ABO gene and various disease risks are well-documented [18-20]. The biological plausibility of these associations is generally explained by the ability of these SNPs to influence the levels of various biological molecules (*e.g*, plasma levels of liver enzymes) and disease-associated traits (*e.g*, hypertension) [18-20]. Similarly, in our study, we found that the ABO rs2073823 polymorphism “A/A” genotype, which has a protective effect on MVC, is associated with lower levels of total cholesterol, LDL-cholesterol, triglycerides, and sP-selectin. These findings suggest that the protective effect of this polymorphism may result from its ability to maintain lipid profile homeostasis and down-regulate endothelial dysfunction. In summary, the rs2073823 polymorphism is added to the previously identified ABO gene polymorphisms, reflecting the gene’s pleiotropy [22, 24-29].

This study was conducted on an Iraqi population. Iraq is an Asiatic and Arabic country where diabetes is prevalent. An exome-wide association analyses study testing the genetic susceptibility to T2D MVC in the United Arab Emirates, a neighboring Arabic country, did not reveal any associations with the ABO gene’s SNPs [12]. This exploratory study provides valuable insights into whether genetic variations in the ABO gene modulate T2D MVC. Additionally, this study included important metabolic, inflammatory, and endothelial biomarkers related to T2D MVC, influenced by genetic variations in the ABO gene. However, further studies should replicate our investigations with designs that address our study’s limitations.

The present study has several limitations. First, the sample size is small compared to previous studies, particularly those examining the pleiotropic effects of the ABO gene. Second, there are a small number of T2D patients without MVC. Third, we could not establish a causal correlation between the studied factors and MVC due to the observational nature of our study and the absence of mechanistic studies underlying the functional effects of the polymorphism. Additionally, we adjusted for several MVC risk factors in our analysis; however, data on tobacco smoking and alcohol use are missing in our study. These factors can modulate MVC risk and affect inflammatory and endothelial markers. Adjusting for smoking and alcohol use would confirm the independent effect of the studied SNP. Furthermore, the lack of a non-diabetic control group weakens the interpretation of inflammatory and endothelial marker responses. Lastly, our result could only be generalized to populations sharing a common ethnic origin and genetic background.

## CONCLUSION

In conclusion, we found for the first time an association between the ABO rs2073823 polymorphism and MVC risk in the Iraqi population. The ABO rs2073823 “AA” genotype and “A” allele may confer a protective effect against MVC and DR, respectively. Furthermore, our results suggest that the protective role of this polymorphism could be due to its ability to modulate blood lipid and sP-selectin levels. Larger studies with a more diverse population are needed to explore this protective effect.

## Figures and Tables

**Fig. (1):**
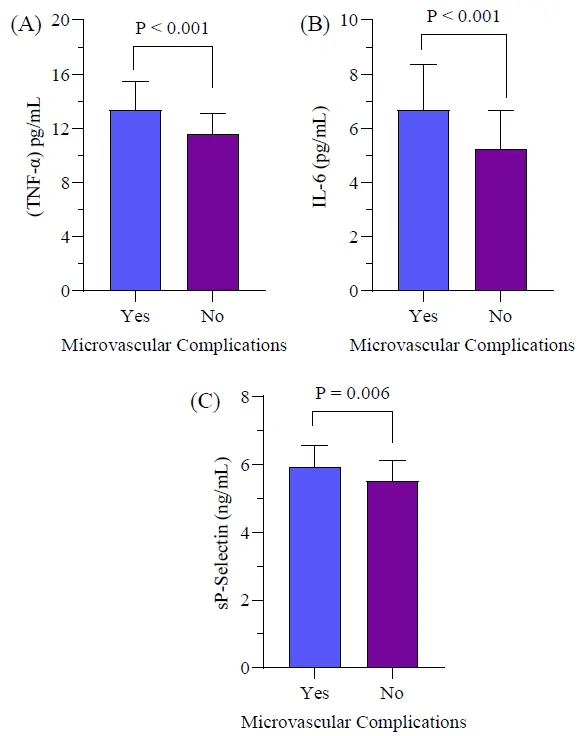
Levels of TNF-α **(Α)**, IL-6 **(Β)** and sP-selectin **(C)** in the study participants stratified by microvascular complications.

**Fig. (2):**
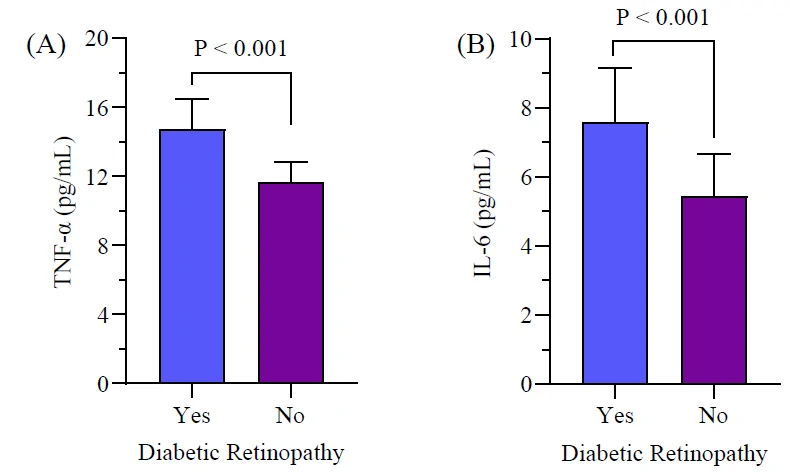
Levels of TNF-α **(A)** and IL-6 **(B)** in the study participants stratified by diabetic retinopathy.

**Fig. (3):**
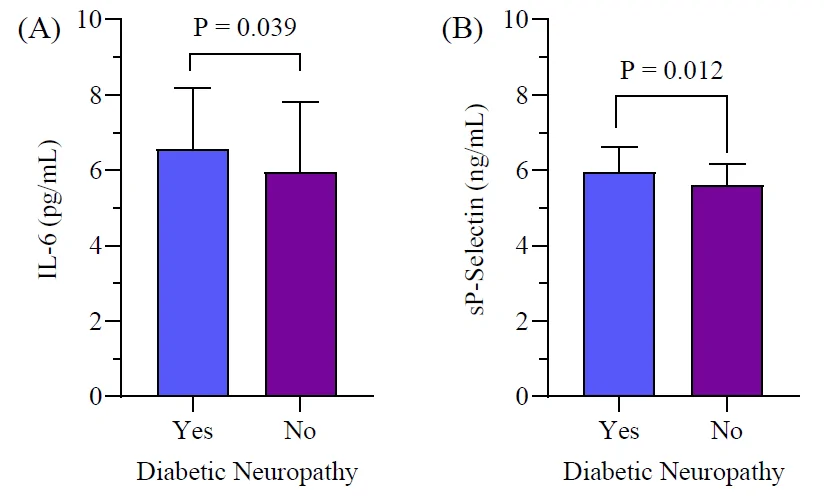
Levels of IL-6 **(A)** and sP-selectin **(B)** in the study participants stratified by diabetic neuropathy.

**Fig. (4):**
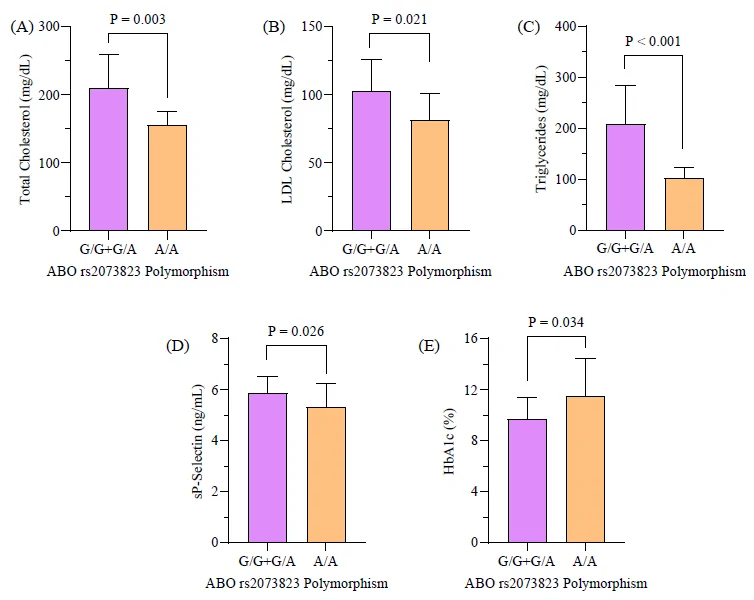
Levels of total cholesterol **(A)**, LDL-cholesterol **(B)**, triglycerides **(C)**, sP-selectin **(D)** and HbA1c **(E)** according to the ABO rs2073823 polymorphism under recessive genetic model.

**Fig. (5):**
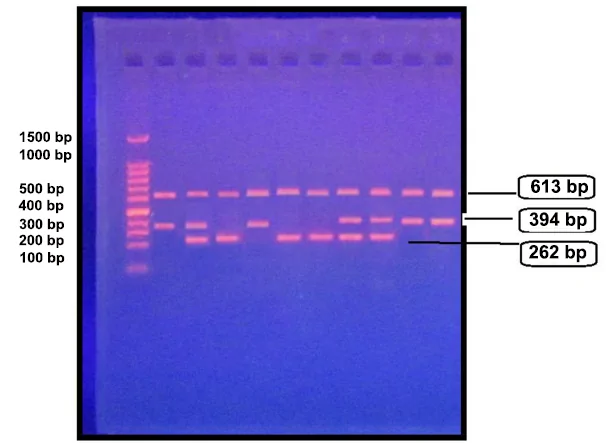
Agarose gel electrophoresis ARMS assay for detection ABO rs2073823 polymorphism. Lane 1: represented DNA marker; Lane 2, 5, 10 and 11 represented wild type; Lane 3, 6 and 7 represented mutant type; Lane 3, 8 and 9 represented heterozygous type.

**Table 3:** Genotypes distribution and allelic frequencies of ABO rs2073823 in patients with microvascular complications and patients without microvascular complications.

**ABO** **rs2073823**	-	**Microvascular complications**		p*	**Unadjusted** **OR (95% CI)**	-	**Multi-adjusted** OR (95% CI)**	***p***	**AIC**	**BIC**
		**Yes** **n=72**	**No** **n=24**							
Genetic model	-	-	-	-	-	-	-	-	-	-
	Genotype	-	-	-	-	-	-	-	-	-
Co-dominant	-	-	-	0.039	-	-	-	-	108.4	116.1
	G/G	50 (69.4)	16 (66.7)	-	1.00 (referent)	-	1.00 (referent)	-	-	-
	G/A	20 (27.8)	4 (16.7)	-	1.60 (0.48-5.38)	0.447	2.54 (0.66-9.72)	0.175	-	-
	A/A	2 (2.8)	4 (16.7)	-	0.16 (0.27-0.96)	0.045	0.14 (0.02-0.91)	0.040	-	-
Dominant	-	-	-	0.799	-	-	-	-	111.9	117
	G/G	50 (69.4)	16 (66.7)	-	1.00 (referent)	-	1.00 (referent)	-	-	-
	G/A+A/A	22 (30.6)	8 (33.3)	-	0.88 (0.33-2.36)	0.799	1.27 (0.40-4.00)	0.688	-	-
Recessive	-	-	-	0.033	-	-	-	-	107	112.1
	G/G+G/A	70 (97.2)	20 (83.3)	-	1.00 (referent)	-	1.00 (referent)	-	-	-
	A/A	2 (2.8)	4 (16.7)	-	0.14 (0.02-0.84)	0.031	0.11 (0.02-0.72)	0.022^¥^	-	-
Over-dominant	-	-	-	0.276	-	-	-	-	110.7	115.8
	G/G+A/A	52 (72.2)	20 (83.3)	-	1.00 (referent)	-	1.00 (referent)	-	-	-
	G/A	20 (27.8)	4 (16.7)	-	1,92 (0.58- 6.33)	0.282	3.51 (0.90-13.7)	0.070	-	-
Allelic	Allele	-	-	0.200	-	-	-	-	-	-
	G	120 (83.3)	36 (75.0)	-	1.00 (referent)	-	-	-	-	-
	A	24 (16.7)	12 (25.0)	-	0.60 (0.27-1.32)	0.203	-	-	-	-

Note: *, *p*-value of χ^2^ tests; **, adjusted by age, gender, body mass index, duration of diabetes and hypertension; ^¥^ *p*-value of best-fit model 95% CI: 95% confidence interval; AIC, Akaike’s Information criterion; BIC, Bayesian Information criterion; OR: odds-ratio.

**Table 4:** Variation in levels of biochemical markers according to the ABO rs2073823 polymorphism under the recessive genetic model.

**-**	**ABO rs2073823**		***p***	**Effect Size**	p*
	**G/G+G/A**	**A/A**			
eGFR	93.6±30.4	102±35.9	0.607	-0.22	0.21 (0.13) ; 0.607
Creatinin	0.76±0.19	0.70±0.24	0.342	0.40	-0.29 (0.11); 0.501
Fasting blood glucose	273±100.37	327±198	0.520	-0.27	0.11(0.16) ; 0.779
HbA1c	9.69±1.72	11.5±2.94	0.049	-0.84	0.78 (0.07) ; 0.034
Total cholesterol	210±49.33	155±21.0	0.009	1.11	-1.22 (0.11) ; 0.003
HDL-cholesterol	37.9±6.10	35.3±8.02	0.298	0.44	-0.62 (0.08) ; 0.155
LDL-cholesterol	103±23.2	81.7±19.4	0.036	0.89	-0.98 (0.11); 0.021
Triglycerides	208±76.3	102±21.5	<0.001	1.83	-1.75 (0.16) ; <0.001
TNF-α	13.0±2.13	12.3±1.03	0.509	0.28	-0.14 (0.06); 0.729
IL-6	6.41±1.72	5.33±1.37	0.108	0.68	-0.61 (0.10); 0.128
sP-Selectin	5.86±0.63	5.33±0.90	0.028	0.94	-0.94 (0.05) ; 0.026
sE-Selectin	1.70±0.36	1.93±0.37	0.126	-0.65	0.83 (0.09); 0.053

**Note: **Data are expressed as mean ± standard deviation;*, *p* value adjusted by age, gender, body mass index, duration of diabetes and hypertension.

**Table 1:** Demographic and clinical characteristics of the study participants in whole and stratified by microvascular complications.

-	**Total** **n=96**	**Microvascular Complications**		***p***	**Effect Size**
		**Yes** **n=72**	**No** **n=24**		
Gender, male	38 (39.6)	30 (41.7)	8 (33.3)	0.470	-
Hypertension	38 (39.6)	32 (44.4)	6 [25]	0.092	-
Age, years	57.1±8.14	58.2±7.47	53.7±9.22	0.012	-0.61
Body mass index, Kg/m^2^	31.3±5.92	31.8±6.00	30.1±5.57	0.211	-0.30
Duration of disease, years	14.1±6.69	14.6±6.40	12.6±7.44	0.107	-0.51
eGFR	94.2±30.6	96.3±31.56	87.8±27.10	0.293	-0.25
Creatinine	0.76±0.19	0.75±0.20	0.80±0.15	0.143	0.35
Fasting blood glucose	276.5±108	273.9±102.9	284.4±124.1	0.815	0.06
HbA1c	9.80±1.85	9.79±1.69	9.85±2.29	0.932	-0.02
Total cholesterol	206.7±49.9	207.1±50.0	205.2±50.4	0.951	-0.01
HDL-cholesterol	37.8±6.21	37.8±6.47	37.6±5.49	0.970	-0.01
LDL-cholesterol	101.9±23.5	100.8±25.3	105.0±16.9	0.295	0.25
Triglycerides	201.4±78.3	206.7±75.3	185.6±86.7	0.137	-0.35

**Note: **Data are expressed as mean ± standard deviation or number (percentage).

**Table 2:** Levels of studied inflammatory markers of the study participants stratified by microvascular complications, diabetic retinopathy and diabetic neuropathy.

-	**Microvascular Complications**		***p***	**Effect Size**	**Diabetic Retinopathy**		***p***	**Effect Size**	**Diabetic Neuropathy**		***p***	**Effect Size**
	**Yes** **n=72**	**No** **n=24**			**Yes** **n=40**	**No** **n=56**			**Yes** **n=62**	**No** **n=34**		
TNF-α	13.4±2.05	11.6±1.47	< .001	-1.00	14.7±1.78	11.7±1.14	< 0.001	-2.04	13.2±1.92	12.5±2.33	0.179	-0.29
IL-6	6.70±1.67	5.25±1.39	< .001	-1.02	7.57±1.60	5.46±1.19	< 0.001	-1.56	6.56±1.61	5.94±1.86	0.039	-0.45
sP-Selectin	5.93±0.64	5.53±0.60	0.006	-0.66	5.85±0.66	5.82±0.66	0.755	-0.06	5.95±0.67	5.61±0.56	0.012	-0.55
sE-Selectin	1.75±0.37	1.61±0.32	0.108	-0.38	1.77±0.36	1.68±0.36	0.188	-0.27	1.77±0.39	1.61±0.29	0.056	-0.41

**Note: **Data are expressed as mean ± standard deviation.

## Data Availability

The data that support the findings of this study are available from the corresponding author.

## LIST OF ABBREVIATIONS

DN: Diabetic Neuropathy
MVCs: Microvascular Complications
T2D: Type 2 Diabetes
AIC: Akaike Information Criterion
BIC: Bayesian Information Criterion
