# Supplementary material for: Association of ABO Gene rs2073823 Polymorphism with Microvascular Complications, sP-Selectin Levels and Lipid Profile in Type 2 Diabetes
Source: Curr Diabetes Rev. 2025 Jan 16;22(2):E15733998341750. doi: 10.2174/0115733998341750241208055924 (PMC13711330; doi:10.2174/0115733998341750241208055924)
Supplement: Supplementary file 1 [file CDR-22-2-E15733998341750_SD1.pdf]

Supplementary Material

Association of ABO Gene rs2073823 Polymorphism with Microvascular Complications, sP-Selectin Levels and Lipid Profile in Type 2 Diabetes

Haithem Rauf Mohammed[1,2], Rym Ben Othman[3,4,*] , Zahraa Saad Hatef[5], Mohamed Kacem Ben Fradj[6] and Haifa Abdesselem[7]

[1]*Faculty of Pharmacy, University of Monastir, Tunisia;* [2]*College of Medicine, University of Karbala, Iraq;* [3]*Faculty of Medicine of Tunis, University of Tunis el Manar, Tunisia;* [4]*Institut National de Nutrition et de Technologie Alimentaire de Tunis, service A, Tunisia;* [5]*Al Safwa University College, Karbala, Iraq;* [6]*Laboratory of Biochemistry, LR99ES11, Rabta University Hospital, Tunis, Tunisia;* [7]*Institut National de Nutrition et de Technologie Alimentaire de Tunis, service D, Tunisia*

Table S1. Primers sequence for ABO rs2073823.

Primers	Accession No.	Sequences
(forward) (outer)	rs 2073823 (G\A)	5'- CTTCCTCCCTCCAGGCTTGA-3'
(reverse) (outer)		5'- AGTGGCGAGTGACTGTGGACA-3'
(forward) (inner)		5'CCCCTCCTCTCTGTAACTGTGTCC-3'
(Reverse) (inner)		5'GTGGCTCAGCATGACGGACA-3'

Table S2. Demographic and clinical characteristics of the study participants stratified by retinopathy.

-	Retinopathy		P	Effect Size
.	Yes	No		
Gender, male	16 (40.0)	22 (39.3)	0.944	-
Hypertension	26 (65)	12 (21.4)	< 0.001	-
Age, years	60.6±7.53	54.6±7.66	< 0.001	-0.76
Body mass index, Kg/m^2	33.1±6.92	30.1±4.76	0.035	-0.46
Duration of disease, years	17.2±6.21	11.9±6.15	< 0.001	-0.84
eGFR	95.2±27.9	93.4±32.6	0.546	-0.13
Creatinine	0.75±0.17	0.77±0.21	0.840	0.04
Fasting blood glucose	254.4±108.6	292.3±105.8	0.048	0.42
HbA1c	9.27±1.50	10.2±1.98	0.025	0.47
Total cholesterol	211.1±50.0	203.5±49.9	0.509	-0.14
HDL- cholesterol	37.4±6.55	38.0±6.01	0.617	0.10
LDL-cholesterol	102.6±26.5	101.4±21.3	0.951	0.01
Triglycerides	219.8±73.0	188.2±79.9	0.018	-0.50

Note: Data are expressed as mean ± standard deviation or number (percentage).

Table S3. Demographic and clinical characteristics of the study participants stratified by neuropathy.

-	Neuropathy		P	Effect Size
	**Yes**	**No**		
Gender, Male	24 (44.4)	10 (33.3)	0.320	-
Hypertension	24 (44.4)	12 (40)	0.693	-
Age, years	57.3±7.41	56.6±9.43	0.579	-0.12
Body mass index, Kg/m^2	31.2±6.11	31.6±5.63	0.785	0.06
Duration of disease, years	14.4±6.34	13.5±7.34	0.173	-0.29
eGFR	98.3±32.6	86.6±25.25	0.121	-0.33
Creatinine	0.74±0.21	0.79±0.15	0.158	0.30
Fasting blood glucose	280.8±105.	268.8±114.04	0.523	-0.14
HbA1c	9.93±1.73	9.58±2.06	0.320	-0.21
Total cholesterol	204.6±50.9	210.4±48.39	0.520	0.14
HDL- cholesterol	37.6±6.47	38.0±5.80	0.737	0.07
LDL-cholesterol	98.9±23.87	107.2±22.23	0.096	0.36
Triglycerides	202.6±71.6	199.3±90.43	0.599	-0.11

Note: Data are expressed as mean ± standard deviation or number (percentage).

Table S4. Genotypes distribution and allelic frequencies of ABO rs2073823 in patients with diabetic retinopathy and patients without diabetic retinopathy.

**ABO rs2073823**		**Diabetic Retionopathy**		**P***	**Unadjusted OR (95% CI)**		**Multi-adjusted OR (95% CI)****	**P**	**AIC**	**BIC**
		**Yes**	**No**							
Genetic model										
	Genotype									
Co-dominant				0.044						
	G/G	32 (80)	34 (60.7)		1.00 (referent)		1.00 (referent)		128	135.7
	G/A	8 (20)	16 (28.6)		0.53 (0.20-1.41)	0.204	1.87 (0.41-8.47)	0.419		
	A/A	0 (0)	6 (10.7)		NC	NC	NC	NC		
Dominant										
	G/G	32 (80)	34 (60.7)	0,044	1.00 (referent)		1.00 (referent)		130.2	135.4
	G/A+A/A	8 (20)	22 (39,3)		0.39 (0.15-0.99)	0.048	0.86 (0.24-3.09)	0.816		
Recessive										
	G/G+G/A	40 (100)	50 (89,3)	0.039¥	1.00 (referent)		1.00 (referent)		127.7	132.8
	A/A	0 (0)	6 (10.7)		NC	NC	NC	NC ¥		
Over-dominant				0.339						
	G/G+A/A	32 (80)	40 (71,4)		1.00 (referent)		1.00 (referent)		133.5	138.6
	G/A	8 (20)	16 (28.6)		0.63 (0.24-1.64)	0.341	2.20 (0.47-10.2)	0.313		
Allelic	Allele			0.009						

	G	72 (90)	84 (75)		1.00 (referent)		-	-		
	A	8 (10)	28 (25)		0.33 (0.14-0.77)	0.011	-	-		

*, P-value of χ2 tests; **, adjusted by age, gender, body mass index, duration of diabetes and hypertension; ¥ P-value of best-fit model 95% CI: 95% confidence interval; AIC, Akaike's Information criterion; BIC, Bayesian Information criterion; NC, not calculable; OR: odds-ratio.

Table S5. Genotypes distribution and allelic frequencies of ABO rs2073823 in patients with diabetic neuropathy and patients without diabetic neuropathy.

**ABO rs2073823**		**Diabetic neuropathy**		P*	**Unadjusted OR (95% CI)**		**Multi-adjusted OR (95% CI)****	**P**	**AIC**	**BIC**
		**Yes**	**No**							
Genetic model										
	Genotype									
Co-dominant				0.155					127.2	134.8
	G/G	42 (67.7)	24 (70.6)		1.00 (referent)		1.00 (referent)			
	G/A	18 (29)	6 (17.6)		1.71 (0.60-4.91)	0.315	2.23 (0.70-7.07)	0.175		
	A/A	2 (3.2)	4 (11.8)		0.29 (0.05-1.68)	0.165	0.34 (0.05-2.23)	0.264		
Dominant				0.774					128.7	133.8
	G/G	42 (67.7)	24 (70.6)		1.00 (referent)		1.00 (referent)			
	G/A+A/A	20 (32.3)	10 (29.4)		1.14 (0.46-)2.84	0.774	1.47 (0.53-4.08)	0.460		
Recessive				0.098					126.2	131.3
	G/G+G/A	60 (96.8)	30 (88.2)		1.00 (referent)		1.00 (referent)			
	A/A	2 (3.2)	4 (11.8)		0.25 (0.04-1.44)	0.121	0.26 (0.04-1.63)	0.151¥		
Over-dominant				0.218					127.2	132.3
	G/G+A/A	44 (71)	28 (82.3)		1.00 (referent)		1.00 (referent)			
	G/A	18 (29)	6 (17.6)		1.91 (0.68-5.39)	0.222	2.56 (0.82-7.97)	0.104		
Allelic	Allele			0.629						
	G	102 (82.3)	54 (79.4)		1.00 (referent)		-	-		
	A	22 (17.8)	14 (20.6)		0.83 (0.40-1.76)	0.629	-	-		

*, P-value of χ2 tests; **, adjusted by age, gender, body mass index, duration of diabetes and hypertension; ¥ P-value of best-fit model 95% CI: 95% confidence interval; AIC, Akaike's Information criterion; BIC, Bayesian Information criterion ;OR: odds-ratio.

Table S6. Variation in levels of biochemical markers according to the ABO rs2073823 polymorphism under the co-dominant genetic model.

-	**ABO rs2073823**			**P**
	**G/G**	**G/A**	**A/A**	
eGFR	94.2±29.5	92.0±33.3	102±35.9	0.748
Creatinine	0.76±0.17	0.77±0.25	0.70±0.24	0.618
Fasting blood glucose	276±101	264±101	327±198	0.720
HbA1c	9.54±1.67	10.1±1.81	11.5±2.94	0.062
Total cholesterol	206±49.8	221±47.3	155±21.0	0.016
HDL- cholesterol	37.4±6.56	39.5±4.32	35.3±8.02	0.187
LDL-cholesterol	103±25.5	104±15.8	81.7±19.4	0.094
Triglycerides	206±81.9	212±59.4	102±21.5	<0.001
TNF-α	13.2±2.32	12.5±1.35	12.3±1.03	0.391
IL-6	6.61±1.79	5.86±1.43	5.33±1.37	0.053
sP-Selectin	5.77±0.61	6.11±0.61	5.33±0.90	0.008
sE-Selectin	1.72±0.33	1.66±0.44	1.93±0.37	0.196

Note: Data are expressed as mean ± standard deviation.
